# Monitoring carbon emissions using deep learning and statistical process control: a strategy for impact assessment of governments’ carbon reduction policies

**DOI:** 10.1007/s10661-024-12388-6

**Published:** 2024-02-03

**Authors:** Chinedu Pascal Ezenkwu, San Cannon, Ebuka Ibeke

**Affiliations:** School of Creative and Cultural Business, Robert Gordon University, Aberdeen, UK

**Keywords:** Carbon emissions, LSTM, Statistical process control, Artificial intelligence, Climate change, Energy policy, Deep learning, ARIMA, Exponential smoothing, ANN

## Abstract

Across the globe, governments are developing policies and strategies to reduce carbon emissions to address climate change. Monitoring the impact of governments’ carbon reduction policies can significantly enhance our ability to combat climate change and meet emissions reduction targets. One promising area in this regard is the role of artificial intelligence (AI) in carbon reduction policy and strategy monitoring. While researchers have explored applications of AI on data from various sources, including sensors, satellites, and social media, to identify areas for carbon emissions reduction, AI applications in tracking the effect of governments’ carbon reduction plans have been limited. This study presents an AI framework based on long short-term memory (LSTM) and statistical process control (SPC) for the monitoring of variations in carbon emissions, using UK annual CO2 emission (per capita) data, covering a period between 1750 and 2021. This paper used LSTM to develop a surrogate model for the UK’s carbon emissions characteristics and behaviours. As observed in our experiments, LSTM has better predictive abilities than ARIMA, Exponential Smoothing and feedforward artificial neural networks (ANN) in predicting CO2 emissions on a yearly prediction horizon. Using the deviation of the recorded emission data from the surrogate process, the variations and trends in these behaviours are then analysed using SPC, specifically Shewhart individual/moving range control charts. The result shows several assignable variations between the mid-1990s and 2021, which correlate with some notable UK government commitments to lower carbon emissions within this period. The framework presented in this paper can help identify periods of significant deviations from a country’s normal CO2 emissions, which can potentially result from the government’s carbon reduction policies or activities that can alter the amount of CO2 emissions.

## Introduction

Climate change is one of the most pressing global environmental issues, with carbon emissions contributing significantly. Due to the urgency of this issue, governments across the world have developed and implemented various policies and plans to reduce carbon emissions. Examples of these efforts include the Paris Agreement (Dimitrov, 2016), the US Environmental Protection Agency’s Clean Power Plan (U.S. Environmental Protection Agency, 2016) and the UK’s Sixth Carbon Budget (Committee on Climate Change, nd). Crucial aspects of these policies include incentivising renewable energy sources, promoting energy efficiency, and implementing carbon pricing mechanisms. Even though these carbon reduction policies can help to reduce future carbon emissions, monitoring their impact is essential but daunting.

Carbon emissions are the product of diverse operations, including manufacturing, transportation, and agriculture. As such, monitoring all of these emissions requires a vast amount of data aggregated from multiple sources. In addition to the difficulty in obtaining these data due to a lack of transparency in the industrial reportage of emissions data (Deane et al., 2017), the monitoring process is complex and requires advanced computations. Technologies such as deep learning (LeCun et al., 2015) and statistical process control (SPC) (Oakland & Oakland, 2018) have evolved as effective computational techniques for data analysis and process monitoring, with applications in several sectors, including manufacturing, healthcare, and finance. This study explores the applications of these technologies in environmental monitoring, considering the impact of governments’ carbon reduction initiatives, using UK annual CO2 emission (per capita) data from 1750 to 2021 (Ritchie et al., 2020).

Recurrent Neural Networks (RNNs) are the most popular deep learning architecture for time series analysis because they can model sequential data, using the output of past time steps as inputs to the current time step (Medsker & Jain, 2001). The feedback connections in RNN and its variants make them suitable for processing audio, videos, and texts, with applications in machine translation (Wu et al., 2016), handwriting recognition (Graves et al., 2008), speech recognition (Zia & Zahid, 2019), robot control (Mayer et al., 2006), and time series analysis (Siami-Namini et al., 2018a; Karim et al., 2017). Standard RNNs struggle with modelling long-term dependencies due to their susceptibility to the vanishing gradient problem. To solve the vanishing gradient issue in RNN, Long Short-Term Memory (LSTM) has been introduced (Hochreiter & Schmidhuber, 1997). LSTMs learn long-dependencies by incorporating a memory cell that selectively retains or forgets information from previous time steps. In contrast to traditional time series models, like autoregressive integrated moving average (ARIMA) model (Shumway et al., 2017), which often require strong pre-existing assumptions about the underlying data distribution and relationships between variables, deep learning techniques such as LSTMs can learn sequential representations without the need for such suppositions, making them effective in modelling complex, non-linear relationships (Siami-Namini et al., 2018b; Karim et al., 2017). Moreover, unlike traditional time series models, which often use seasonal dummies to capture the effect of seasonality, including annual seasonality, ANN, such as LSTM models, do not typically use dummies for seasonal effects, as they can capture seasonal patterns implicitly (Zhang & Qi, 2005; Heshmatol Vaezin et al., 2022).

In this study, we first compared the performances of LSTM, ARIMA, Exponential Smoothing (Ostertagová & Ostertag, 2011) and feedforward ANN (Sazli, 2006) in predicting CO2 emissions on a yearly prediction horizon. Due to its superior performance compared to other models, LSTM was selected for developing a surrogate model of the UK’s carbon emissions characteristics and behaviours based on the experiment’s outcomes. Using SPC, specifically the Shewhart individual-moving range (I-MR) control chart, we evaluate the variations and trends in these behaviours using the deviations of the recorded emission data from the surrogate process. SPC is a statistical technique that can provide insight into the variability within a process. With SPC techniques, it is possible to spot and interpret anomalies or unusual changes in the emissions data. The combination of deep learning and SPC, which has successfully been used in analysing SCADA data associated with wind turbines (Udo & Muhammad, 2021), can provide an effective tool for monitoring the impact of the efforts by the UK government to reduce carbon emissions.

The contributions of this paper can be summarised as follows:

- Available research publications in this area demonstrate that this paper is the first to apply a hybrid technology, consisting of LSTM and SPC, to carbon emissions monitoring, using LSTM to model the baseline behaviours of UK carbon emissions (per capita) and SPC to detect assignable variations.
- This paper is also the first to discuss the control chart obtained from applying computational and statistical process techniques to $CO_2$ emission data in line with known UK government carbon reduction commitments.

These contributions are vital to monitoring the effectiveness of the government’s carbon reduction policies, which are crucial in combating climate change. By continuously evaluating the outcomes, we can identify effective strategies and pinpoint areas that need improvement to ensure that the policies align with the government’s climate objectives towards a sustainable and low-carbon future.

## Review of related literature

Several researchers have successfully applied artificial intelligence and machine learning to forecast carbon emissions, supporting the development of effective environmental policies for reducing carbon emissions. Acheampong and Boatang used ANN in training models for forecasting the intensity of carbon emissions in Australia, Brazil, China, India, and the USA with minimal error (Acheampong & Boateng, 2019). Their study selected nine crucial parameters contributing to carbon emissions intensity as input variables, including economic growth, energy consumption, R &D, financial development, foreign direct investment, trade openness, industrialisation, and urbanisation. The ANN models were validated and can be used by international organisations and environmental policymakers to forecast and make climate change policy decisions.

Agbulut proposed a framework relying on three machine learning algorithms — deep learning, support vector machine(SVM), and ANN — to forecast energy consumption and CO2 emissions relating to Turkey’s transportation sector (Ağbulut, 2022). The study used gross domestic product per capita, population, vehicle kilometres, and year as inputs. It concluded that policymakers need future energy investments to establish regulations, policies, norms, restrictions, legislations, and initiatives to mitigate energy consumption and emissions from the transportation sector.

Dozic and Urosevic (2019) examined an ANN model of the EU’s energy system, which predicts CO2 emissions until 2050, considering the current Energy Policy of the EU (Dozic & Urosevic, 2019). The study concluded that the model is highly effective in predicting the behaviour of CO2 emissions. It can facilitate timely corrections to energy and economic strategies by adjusting relevant indicators to meet the ambitious CO2 emission reduction targets set by the Energy Roadmap 2050 document of the European Commission. Their research analysed several ANN structures to identify the most effective model for large energy systems.

Huang (2021) contributed to China’s national policy plan for achieving a carbon peak in the mid-to-long term, focusing on the Yangtze River Economic Belt basin (Huang et al., 2021). The author’s goal was to comprehensively promote energy conservation and reduce emissions using a hybrid model of LSTM and support vector regression (SVR) to manage and forecast carbon emissions. The model in their research uses information indicators such as industry investment, labour efficiency output, and carbon emission intensity to predict carbon emissions accurately. Other researchers have employed schemes based on SPC to monitor and recommend reducing carbon emissions.

Shamsuzzaman et al. (2021) developed a technique for monitoring carbon emissions from the industrial sector using SPC (Shamsuzzaman et al., 2021). The authors introduced an economic-statistical design for the combined Shewhart $\bar{X}$ and exponentially weighted moving average (EWMA) scheme, which can help to monitor carbon emissions for prompt action to control excessive emissions. The proposed Statistical Process Monitoring (SPM) scheme parameters have been optimised to minimise the total cost, including carbon emissions and operational costs. Actual data from different industrial facilities have been used to demonstrate the application of the proposed SPM scheme and its effectiveness in reducing costs associated with excessive carbon emissions from industries.

Although the above papers demonstrate excellent applications of AI or SPC in carbon emission monitoring or control, their results suffer limitations associated with these techniques. For example, while ANNs can learn complex non-linear patterns and relationships in time series data, unlike SPC, they cannot effectively monitor and control a process to ensure it operates within specified limits. ANNs are better suited for predictive modelling and forecasting, while SPC is better for monitoring and control. This paper proposes a hybrid technique consisting of LSTM and SPC. LSTM can be used to model carbon emission characteristics from historical carbon emission data. At the same time, SPC can identify whether this process entails a natural or a caused variation.

## Methodology

### Data description

The data used for this research is the UK annual CO2 emission (per capita) data, covering between 1750 and 2021 (Ritchie et al., 2020). Figure 2a presents the raw data. The records are based on production or territorial emissions from burning fossil fuels or cement production within the UK’s borders and do not include emissions from traded goods. Moreover, the numbers are specific to CO2 emissions, not total greenhouse gas emissions. Table 1 presents the descriptive statistics of the dataset. As can be seen, the data is continuous, negatively skewed, and platykurtic.

### The workflow

**Fig. 1:**
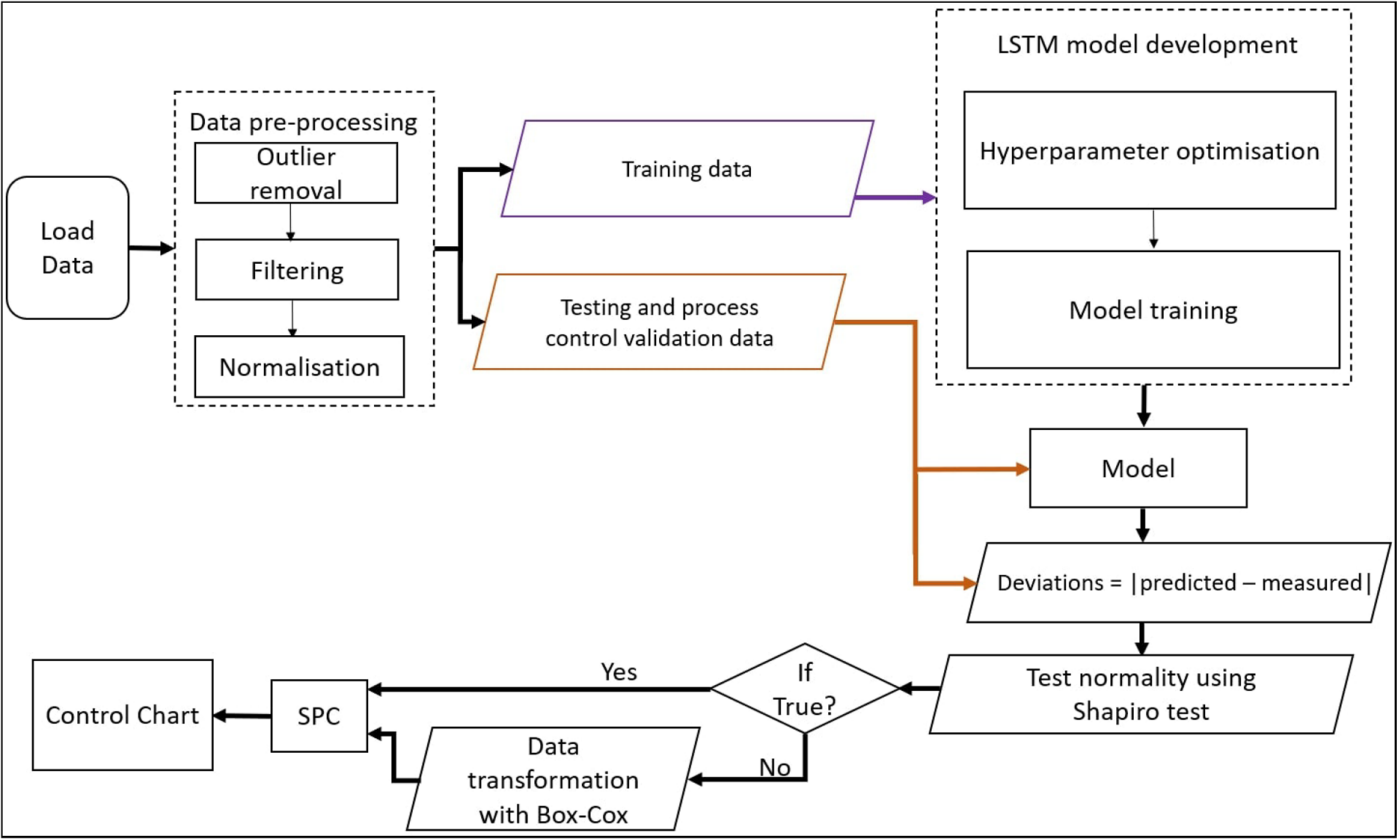
Research workflow

Figure 1 presents the workflow involving the techniques developed for this research.

#### Data pre-processing

This phase involves outlier removal, filtering, and normalisation. This paper applies isolation forest (Liu et al., 2008) for outlier detection and removal. Isolation forest can detect outliers by scoring how easy it is to isolate a single data point from the rest of the data points using a binary search tree. The higher the number of splits required to isolate a data point, the less likely the data point is identified as an outlier.

**Table 1:** Descriptive statistics

Statistic	Value
Count	227.000000
Mean	7.471925
Standard deviation	3.213397
Minimum	1.006713
Kurtosis	−1.139382
Skewness	−0.626540
Median	8.912930
Maximum	11.818837

Filtering, specifically moving averages, follows the outlier removal process to further remove noise from the data and to replace missing values with the mean of their five nearest neighbours. This step is relevant in filtering out false signals, which can obscure the underlying trend in the data and consequently affect the computation of the control limits. The data undergoes z-score normalisation, scaling it down to the interval [0,1] to ensure that the models have consistent scale and distribution, contributing to the efficiency of the learning algorithm.

#### Model development

The initial phase of the study involves evaluating the predictive accuracy of four distinct models on the UK annual CO2 emissions: LSTM, ARIMA, Exponential Smoothing, and Feedforward ANN. The accuracy of the surrogate model is essential for minimising the potential interference of the model inaccuracy with the CO2 emissions monitoring process. The dataset is partitioned into 80% training and 20% testing subsets for the analysis. The training data encompasses annual carbon emissions per capita between 1803 and 1976, while the test data spans from 1977 to 2021.

Among these models, LSTM, ANN, and ARIMA leverage data from the previous three years to predict CO2 emissions for each year, whereas Exponential Smoothing relies on immediate past values for prediction. As a first step towards developing a framework for accurately identifying variations in CO2 emissions within the UK, the goal of the model development process is to effectively represent the typical pattern in the UK’s annual carbon emission data. By utilising SPC, this model can then be used to detect out-of-control situations.

To achieve this aim, the predicted value is subsequently compared with the actual value for the corresponding timestamp, allowing for monitoring changes in CO2 emissions. For example, when predicting the CO2 emissions for 1977, the actual emissions data from 1974 to 1976 is used as input. The disparity between the predicted and actual values is calculated and can be leveraged to monitor fluctuations in CO2 emissions, and this process continues throughout. This approach aligns with the research goal, which is not long-term forecasting of UK CO2 emissions but tracking assignable variations within the emission data.

Hyperparameters for the LSTM, ARIMA, Exponential Smoothing, and ANN were selected using Bayesian Optimisation (Frazier, 2018) available in hyperopt library (Bergstra et al., 2013). Table 2 presents the hyperparameters for models.

**Table 2:** Hyperparameters

Model	Hyperparameter	Value
LSTM	LSTM_1 units	128
	Activation_1	Relu
	LSTM_2 units	64
	Activation_2	Relu
	Dropout	0.2
	Optimizer	Adam
	Learning rate	0.00001
	Loss function	Mean squared error
	Epochs	250000
	Batch size	8
	Validation split	0.2
ARIMA	Autoregressive order (p)	3
	Differencing order (d)	4
	Moving Average order (q)	9
Exponential	Damping factor	0.875
Smoothing		
ANN	Learning rate	0.2071
	Number of hidden neurons	4
	Momentum term	0.0797
	Maximum iteration	830
	Activation	Relu

#### Monitoring the carbon emissions process

The actual monitoring of the carbon emissions process follows the successful model development. Using the data from 1977 to 2021, set aside for model testing and process monitoring, the surrogate model predicts each year’s carbon emission per capita. The absolute deviation of the measured emission from the predicted emission for the year *k* is calculated as follows: $\delta _k = |predicted_k - measured_k|$

Although SPC approaches have been developed for non-normal data, researchers have demonstrated that serious errors can occur in results from non-normal data (Chou et al., 1998; Andrássyová et al., 2012; Xiao et al., 2020). To avoid poor results due to non-normal data, the Shapiro-Wilk test of normality is first used to identify if the deviations are normally distributed or not (Shapiro & Wilk, 1965). The null hypothesis of the Shapiro-Wilk test is that the sample comes from a normally distributed population. The test statistic is calculated as follows:

1 $$\begin{aligned} W = \frac{(\sum _{i=1}^{n} a_i \delta _{(i)})^2}{\sum _{i=1}^{n} (\delta _i - \bar{\delta })^2} \end{aligned}$$

where *n* is the sample size, $\delta (i)$ is the $i-th$ order statistic (i.e., the *ith* smallest value in the sample), $\bar{x}$ is the sample mean, and $a_i$ are constants that depend on *n* and the chosen level of significance. The constants are chosen so that the expected value of *W* is approximately equal to 1 for normal data. The Shapiro-Wilk test compares the value of *W* to critical values obtained from a Shapiro-Wilk critical values table. If the calculated value of *W* is less than the critical value, then the null hypothesis is not rejected, and the sample is considered consistent with normality; otherwise, the null hypothesis is rejected, and the sample is considered to be non-normal.

To avoid challenges posed by non-normal data, the deviations undergo Box-Cox transformation (Box & Cox, 1964) before the SPC process if they are non-normally distributed. The Box-Cox equation is given by

2 $$\begin{aligned} y^{(\lambda )} = {\left\{ \begin{array}{ll} \frac{y^\lambda - 1}{\lambda } &{} \text {if } \lambda \ne 0\\ \ln (y) &{} \text {if } \lambda = 0 \end{array}\right. } \end{aligned}$$

$y^{(\lambda )}$ is the transformed variable; *y* represents the original variable; and $\lambda$ is the transformation parameter. The value of $\lambda$ can be any real number but is often bounded within a range of values depending on the context and the nature of the data. For example, $\lambda$ must be positive if *y* is strictly positive. $\lambda$ is selected to maximize the log-likelihood function to find the best transformation for the data.

Next, SPC can help to investigate regions along a time series to determine if natural or special variations drive them. Natural variations are inherent to a process and are caused by random factors, while special variations are non-random and driven by specific factors, such as a government’s carbon reduction policy, as in the case of this research. To investigate the deviations between the recorded carbon emissions and the value predicted using the surrogate model and to identify the nature of the cause of the deviation for each specific period, we have employed SPC. Specifically, the Shewart control chart (the individual/moving-range (I-MR) chart) has been used to evaluate the deviations over time. I-MR-chart combines the moving range (MR) and the individual control charts in determining the out-of-control situations within a process. Each chart is based on two control limits, the Upper Control Limit (UCL) and Lower Control Limit (LCL), to assess the variations within the data. The control limits establish the chart’s sensitivity to variations within the data points. MR of the deviation distribution, ${\{\delta _i\}}_{i=1}^{m}$, is estimated as the absolute difference between the $i-th$ deviation and its predecessor, the $(i-1)th$.

**Fig. 2:**
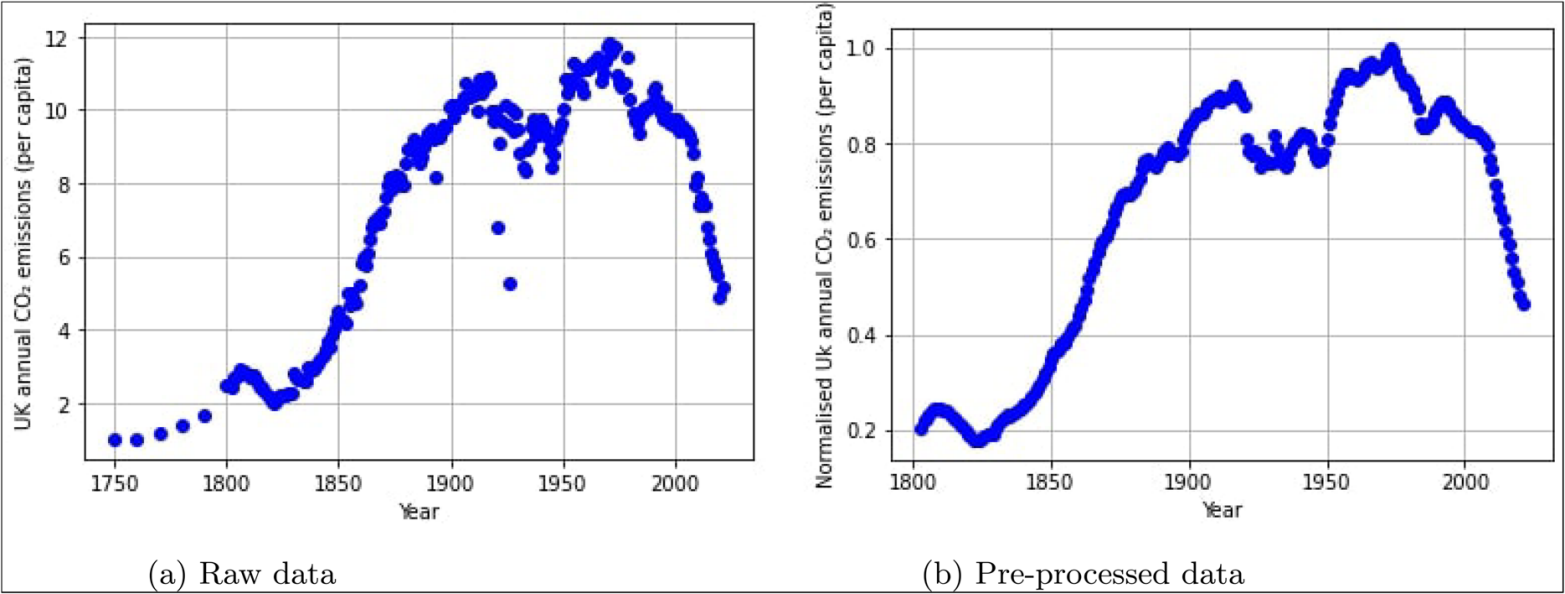
UK annual CO2 emission (per capita) data

The process of computing the control limits for MR is as follows:

- The difference between a data point $\delta _i$ and its predecessor $\delta _{i-1}$ is given by
  3 $$\begin{aligned} MR = |\delta _i - \delta _{i-1}| \end{aligned}$$
- The centre line is computed as the arithmetic mean of the values obtained from step 1 above as follows:
  4 $$\begin{aligned} \displaystyle \overline{MR} = \frac{\sum _{i=1}^{m-1}MR_i}{m-1} \end{aligned}$$
- Calculate control limits
  5 $$\begin{aligned} \displaystyle UCL= & {} D_4*\overline{MR} \end{aligned}$$
  6 $$\begin{aligned} \displaystyle LCL= & {} D_3*\overline{MR} \end{aligned}$$
- Using these values, plot the control chart and provide interpretations.

For the individual chart, the control limits are computed as follows:

- Centre line
  7 $$\begin{aligned} \displaystyle \overline{x} = \frac{\sum _{i=1}^{m}\delta _i}{m} \end{aligned}$$
- Control limits
  8 $$\begin{aligned} \displaystyle UCL= & {} \displaystyle \overline{x} + 3\displaystyle \frac{\overline{MR}}{d_2} \end{aligned}$$
  9 $$\begin{aligned} \displaystyle LCL= & {} \displaystyle \overline{x} - 3\displaystyle \frac{\overline{MR}}{d_2} \end{aligned}$$

where $d_2$, $D_3$, and $D_4$ are anti-biasing constants, with values as 1.128, 0, and 3.267, respectively, being the recommended factors for sample size, *n* = 2 (Montgomery, 2020).

## Results and discussions

### Data cleaning and transformation

Figure 2a and b demonstrate the improvements achieved in the data after passing it through the pre-processing pipeline. The data points before 1800 were considered outliers and were deleted from the dataset. As well as smoothing out and removing noise from the dataset, the moving average is also used to replace missing values. The data is then normalised to the scale [0,1] to ensure that the models have consistent scale and distribution.

### Evaluation of the surrogate model

**Fig. 3:**
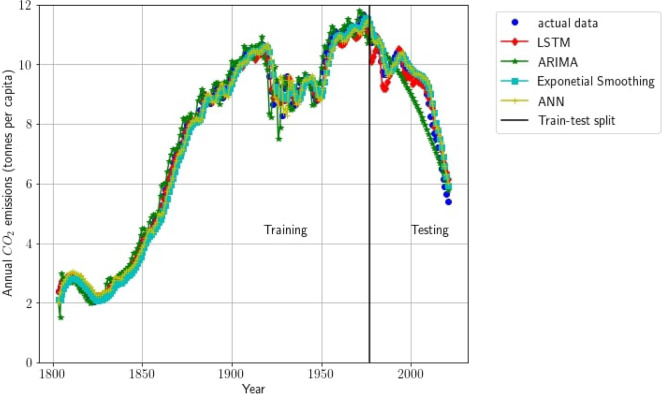
Models’ performance

Figure 3 presents the performance of the models on the data. The first part of the figure showcases how well the models perform on the training subset, while the second part depicts their ability to predict the next CO2 emissions using values from the past three years. Metrics such as mean square error (MSE), root mean square error (RMSE), mean absolute error (MAE), and R-squared are used to evaluate the models and are summarised in Table 3. The results show that the LSTM outperforms the other models while the ARIMA performs the worst. Due to its superior performance, the LSTM is selected as the surrogate model for representing the UK carbon emissions during the process monitoring phase. The accuracy of the surrogate model is paramount in reducing the potential interference of model inaccuracy with the CO2 emissions monitoring process.

### Process monitoring using SPC

**Fig. 4:**
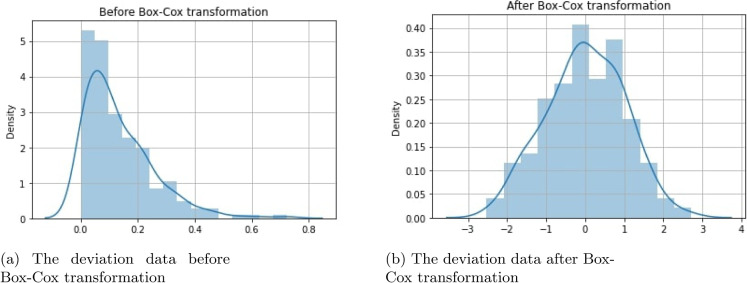
Box-Cox transformation of the deviation data

The absolute difference (or deviation) between the actual UK annual carbon emissions (per capita) and the predicted emissions is first calculated across the time series for the monitoring process. The Shapiro-Wilk normality test demonstrates that the data significantly deviates from a normal distribution with *p*-value ($$= 7.997 \times 10^{-13})<0.05$$. Applying the Box-Cox transformation to the deviation data significantly produced a normally distributed output, with the significance value of the Shapiro-Wilk test, *p*-value($$=0.596 > 0.05)$$. Figure 4 demonstrates the data distributions before and after applying the Box-Cox transformation.

**Fig. 5:**
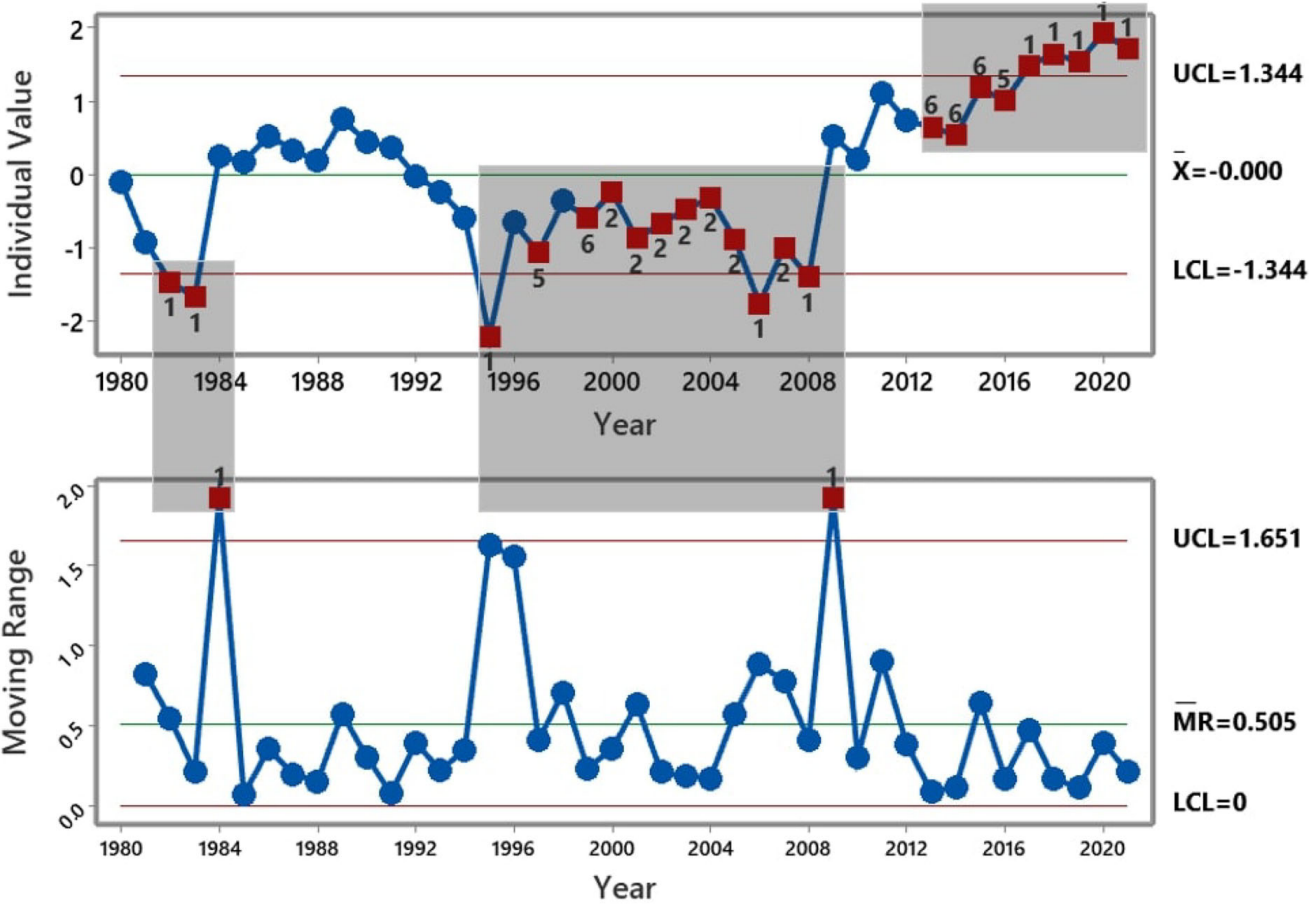
I-MR Charts of the absolute deviation between the actual and predicted carbon emissions values

Figure 5 presents I-MR control charts obtained from the absolute difference between the model predictions and the recorded UK carbon emissions. Following Nelson’s rules for control chart interpretations (Nelson, 1984, 1985), the data points presented in red have been identified as “out-of-control” situations (or assignable causes or special cause variations). Unlike the common cause variations (i.e., data points in blue), which are the natural variations within a system, assignable causes are unexpected. They are often due to external reasons. SPC aims to eliminate assignable variations in several processes, including manufacturing, production, asset management, and service delivery, because they imply a deviation from predictable or known behaviours. However, for a process that seeks to introduce a departure from existing practice, assignable causes could be desirable because they can represent the effect of the actions introduced to cause the change. An example of the situation above where assignable causes can portray a positive change is the effect of a government’s carbon reduction plan on carbon emissions, which is the thesis of this paper. Below are the descriptions of Nelson’s eight rules and their general practical insights:

**Table 3:** Performance scores of the model

Metric	LSTM	ARIMA	Exponential smoothing	ANN
MSE	0.00044	0.2643	0.0249	0.0737
RMSE	0.020	0.211	0.158	0.272
MAE	0.016	0.403	0.125	0.190
$R^2$	0.997	0.971	0.997	0.993

- **Rule 1:** One point is over three standard deviations from the mean — an unusual event or a measurement error.
- **Rule 2:** Nine (or more) points in a row are on the same side of the mean — a slight shift from the average.
- **Rule 3:** Six (or more) points in a row continually increase (or decrease) — a trend pattern.
- **Rule 4:** Fourteen (or more) points alternate in direction, increasing then decreasing — an over-control pattern.
- **Rule 5:** Two (or more) out of three points in a row are more than two standard deviations from the mean in the same direction — a significant shift from the average.
- **Rule 6:** Four (or more) out of five points in a row are more than one standard deviation from the mean in the same direction — a slight shift from the average.
- **Rule 7:** Fifteen points in a row are all within one standard deviation of the mean on either side of the mean — stratification nature of the process.
- **Rule 8:** Eight points in a row exist, but none within one standard deviation of the mean, and the points are in both directions from the mean — a mixture property of the process.

The numbers on the red data points in Fig. 5 indicate the rules used to confirm the points as out-of-control. In the individual (I) and the moving range (MR) charts, only rules 1, 2, 5 and 6 have been violated. Combining I-chart and MR-chart provides a clear picture of the process behaviours using these rules. I-charts can identify any common or assignable causes within a process by monitoring the mean and shifts in the process. In contrast, MR charts monitor the process variations by tracking the absolute difference between known and measured behaviours of the system. Out-of-control situations due to the violation of rule 1 have been highlighted on the I-chart (in 1982,1983,1995,2006,2008, and 2017–2021) and the MR-chart (in 1984 and 2009). Violation of rule 1 can be interpreted as the occurrence of an unusual event or an erroneous measurement of data. Deviations from the then-existing pattern in the UK carbon emissions (per capita) have been recorded between 1997–2004 and 2012–2016 and 2000, as highlighted by the data points numbered 2, 5, and 6 in the I-chart, illustrating violations of the corresponding rules. The control charts reveal that activities that impacted the UK’s carbon emissions per capita intensified from the mid-1990s to 2021.

In line with the observations from the control charts, according to a technical report from the European Environment Agency, between 1990 and 2012, greenhouse gas emissions in the EU decreased, with Germany and the UK accounting for 50% of the EU’s net decrease in emissions within this period (Agency & Agency, 2015). The UK’s main contributor was the liberalisation of energy markets and the subsequent switch from oil and coal to gas as a fuel for electricity production (Agency & Agency, 2015).

Moreover, the intensification of the UK’s commitments towards carbon reduction from the 1990s follows its choice of 1990 as a baseline year for carbon emissions reductions. This baseline commitment choice was primarily due to the United Nations Framework Convention on Climate Change (UNFCCC), established in 1992 but became effective in 1994 (Bodansky, 1993; Greene, 2000). The convention aimed to stabilise greenhouse gas concentrations in the atmosphere at a level that would prevent dangerous anthropogenic interference with the climate system. The developed nations agreed to execute national strategies for tackling climate change to lower anthropogenic greenhouse gas emissions to levels observed in a baseline year.

By setting the baseline year at 1990, the UK committed to reducing its emissions to levels below that year’s emissions (Kelly et al., 2014; Barrett et al., 2018) through several schemes, including the Paris Agreement and the Kyoto Protocol, involving the first and second commitments, covering the periods 2008–2012 and 2013–2020 respectively. Since then, the UK has set several emissions reduction targets, including achieving net zero emissions by 2050 (Pye et al., 2017). Using 1990 as a baseline year, the UK can track its progress towards these targets and monitor its success in reducing its contribution to global greenhouse gas emissions.

We suspect the natural variation recorded between 2009 and 2013 is part of the response to the measures preceding this period, including the first Kyoto Protocol commitment, which could normalise as part of the baseline. However, the Second Kyoto Protocol commitment and several other efforts introduced a shift from the baseline in 2013, leading to caused variation, as seen on the control chart.

#### Correlating the UK government’s known carbon reduction/energy policies and emissions-related events with the out-of-control periods

The control chart’s out-of-control periods (i.e., the shaded region) show correlations with the most significant UK carbon reduction and energy efficiency commitments and plans and events relating to carbon emissions over the years. To demonstrate that the approach in this paper can identify where carbon-related policies and events within the UK may impact its usual carbon emission process, we have identified carbon-related policies and events recorded within the shaded periods. Significant carbon reduction policies and events in the UK that correlate with the shaded regions in the control chart have been presented as follows:

#### 1982–1984

a While no carbon reduction policy or legislation was directly established by the UK government within this period, an earlier policy, such as the UK Energy Conservation Act 1981 (Legislation.gov.uk, 1981), could have affected the CO2 emissions within this period. The Act required energy audits and efficiency measures for public sector buildings and large companies. Its goal was to reduce energy consumption, improve energy efficiency, and promote sustainable development in the UK. Data published by the UK National Infrastructure Commission shows that total inland coal consumption in the UK decreased from 1981 to 1982 by 6.25%.^1^
b A major event within this period, which could impact UK carbon emissions, was the UK miners’ strike (from March 84 to March 85) (Adeney & Lloyd, 2021), which led to the closure of many coal mines in the UK. This closure could decrease carbon emissions around this period since coal significantly contributes to carbon emissions. Mamurekli demonstrated that as well as the reduction in the UK’s coal supply between 1984 and 1985, the UK’s coal consumption reduced from 34.6% of the total energy consumption in 1978 to 25% in 1984–85 (Mamurekli, 2010).

#### 1995–2009

a The liberalisation of the energy market in the UK began in the late 1990s (Stanford, 1998) and paved the way for competition in the generation and supply of electricity. The subsequent “dash for gas” in the 1990s saw a significant increase in the use of natural gas for power generation (Spooner, 1995). This refers to a transition among newly privatised electricity companies in the UK towards generating electricity using natural gas. The “dash for gas” caused a decrease in gas prices, a substantial increase in gas-fired power generation capacity, significant improvements in the average efficiency of gas-fired power plants, and a corresponding rise in total gas-fired electricity generation from 4 TWh in 1990 to 140 TWh in 2003 (Graus et al., 2007). Richardson and Chanwai confirm that the “dash for gas” contributed to reducing the UK’s carbon emissions within this period (Richardson & Chanwai, 2003).
b The UK government levies a fee on the energy used by industry, farms, and the governmental sector. This fee is known as the Climate Change Levy (CCL) (Pearce, 2006). The programme was first implemented in 2001 to promote energy efficiency and lower greenhouse gas pollution, with plans to cut annual emissions significantly by 2010. Since then, it has incentivised businesses to reduce energy consumption, increase the use of renewable energy, and generate government revenue, but it has also increased costs for businesses. Data is needed to conclude how much this scheme contributed to the variability in the UK’s carbon emissions at the outset before it became part of the baseline.
c In 2005, the European Union created the EU Emissions Trading System (EU ETS) as a cap-and-trade programme to lower greenhouse gas emissions from industrial areas (Action, 2013). It limits the overall quantity of emissions that industries can release and covers all EU members, including the UK before it leaves the EU. Companies included in the programme are given permits to cover their emissions. They can purchase or trade these allowances on the market to generate revenue, providing an incentive to cut emissions. Similar to the situation with the CCL, data is needed to conclude how much this scheme contributed to the variability in the UK’s carbon emissions at the outset before it became part of the baseline.
d Energy Performance Certificates (EPCs) were introduced in the UK in 2007 (Watts et al., 2011), a significant move towards increasing building energy efficiency and lowering carbon pollution. EPCs offer details on a building’s energy efficiency and suggestions for development, assisting in spreading knowledge about energy efficiency and encouraging homeowners and sellers to invest in energy-saving technologies.
e Following the Climate Change Act of 2008, the UK government ratified the Kyoto Protocol and committed to reducing greenhouse gas pollution significantly by 2050 (Skiba et al., 2012). In response, the UK has taken measures to support the use of renewable energy, improve the energy economy, and promote low-carbon transit to meet this goal. For example, the UK has established legally binding carbon budgets, passed the Climate Change Act, and committed to providing international climate finance to support developing countries’ climate action. These were targeted at reducing UK’s greenhouse gas emissions by 12.5% below 1990 levels by 2008–2012, a target it had exceeded in 2014 (of Energy & Change, 2015).
f A carbon budget, or cap on the amount of greenhouse emissions the UK can release over five years, was established by the Carbon Budgets Order 2009 (UK Government, 2023a) as a piece of UK law. The UK government adopted policies and steps to decrease emissions and provide regular updates on its progress towards achieving these goals.

#### 2013–2021

a To promote energy efficiency and lower greenhouse gas pollution, the UK passed the Energy Act 2013 into legislation (UK Government, 2023b). It consists of several measures, including the Carbon Price Floor, Electricity Market Reform, Green Deal, Minimum Energy Efficiency Standards, and Renewable Heat Incentives. These regulations seek to advance the use of low-carbon technologies, foster the growth of green energy sources, and improve the energy economy of residential and commercial buildings.
b The Carbon Reduction Commitment(CRC) Energy Efficiency Scheme was a mandatory UK government initiative introduced in 2010 to improve energy efficiency and reduce carbon emissions (UK Department of Energy and Climate Change, 2010; Committee on Climate Change, 2010). However, the CRC Energy Efficiency Scheme was criticised for its complexity, which made compliance challenging and expensive. The scheme was reformed in 2013 to simplify the process, focus on energy efficiency and introduce a performance league table to encourage transparency and improvements. It was later replaced by the Streamlined Energy and Carbon Reporting (SECR) framework in 2019 (UK Government, 2021b).
c The UK government launched the Clean Growth Strategy in 2017 to promote economic growth while reducing greenhouse gas emissions and addressing climate change (Ward & Matikainen, 2018). The strategy outlines various measures to achieve this, including improving energy efficiency in homes and businesses, encouraging the use of low-emission vehicles and investing in infrastructure, supporting the development of low-carbon industries, investing in research and development for new low-carbon technologies, and incentivising businesses to reduce their carbon footprint.
d The UK government and the offshore wind industry launched the offshore wind sector deal in 2019 to significantly increase offshore wind power generation (BEIS, 2019). Its goal is to increase the UK’s offshore wind capacity by 2030 and expand the number of jobs in the sector while contributing to efforts to combat climate change and reduce greenhouse gas emissions. The deal includes strategies such as investment in new offshore wind farms, improvements to supply chains and infrastructure, and support for innovation and research and development.
e The UK government committed in 2019 to achieve net zero carbon emissions by 2050, aiming to limit global warming to 1.5^∘^C above pre-industrial levels and prevent the worst impacts of climate change (UK Government, 2021a). This target is enshrined in law, making the UK the first major economy in the world to commit to net zero carbon emissions by 2050. Strategies include increasing renewable energy generation, phasing out petrol and diesel cars, improving energy efficiency in buildings, and investing in new technologies.
f The COVID-19 pandemic significantly impacted worldwide carbon emissions (Mehlig et al., 2021). With lockdowns and travel restrictions, energy demand was significantly decreased, particularly from transportation and industry. As a result, carbon emissions in the UK fell to their lowest levels in decades, with a 13% reduction compared to the previous year.
g The UK government introduced the Sixth Carbon Budget in December 2020, aiming to achieve the country’s net zero emissions objective by 2050 by lowering greenhouse gas emissions by 78% by 2035 compared to 1990 (UK Government, 2021c). The plan outlines sector-specific emissions reduction goals and methods for achieving them, including growing renewable energy sources, enhancing the energy economy, and utilising fewer fossil fuels for transportation. The UK government accepted the Committee on Climate Change’s proposals and plans to propose legislation to formalise the goals.

## Conclusions and recommendations

This research demonstrates the application of a hybrid technology comprising deep learning and statistical process control in monitoring the impact of the government’s carbon reduction policy on carbon emissions within the UK economy. We first developed the surrogate model of the carbon emissions process of the UK and computed the deviation of out-of-sample measured data from the model. I-MR was employed to identify regions of special cause variations, which we demonstrated to correlate with significant carbon reduction policies of the UK government and known events, such as COVID-19, that can impact UK carbon emissions. However, there are still aspects of this work that warrant future research. For example, it can be challenging to identify each policy’s or event’s contributions to an out-of-control region. Also, we cannot demonstrate whether the responses on the control charts emanated from the long-term or short-term effects of policies. Solving these problems will make it possible to investigate the impact of individual policies and how long they take to reflect on the process. In our future related work, we aim to explore explainable AI applications on this task, leveraging explicit dummies to understand better the influence of policies of interest on carbon emissions data. This paper considers the government’s carbon reduction policies and events such as COVID-19; however, several other events can impact carbon emissions. These activities include economic development, technology, agriculture, and imports. Investigating the impact of changes in the actions within these activities will be a valuable further contribution to knowledge. Although our method cannot recommend future climate policies, when used in combination with a qualitative approach it can be helpful in identifying the impact of existing policies and determining which ones to reinforce for more effective CO2 emissions control.

## Data Availability

The data source for this research has been cited in this paper; it is publicly available on ‘Our World In Data’ https://ourworldindata.org/co2/country/united-kingdom.

## Footnotes

1 https://nic.org.uk/app/uploads/Historical-Energy-Data-Final-Dataset.xlsx
